# Effective Recruitment Strategies for Community-Dwelling Persons Living With Dementia and Their Caregivers

**DOI:** 10.1093/geroni/igab046.2093

**Published:** 2021-12-17

**Authors:** Miranda McPhillips, LaShauna Connell, Augustine Boateng, Darina Petrovsky, Justine Sefcik, Nancy Hodgson

**Affiliations:** 1 University of Pennsylvania, University of Pennsylvania, Pennsylvania, United States; 2 University of Pennsylvania, Philadelphia, Pennsylvania, United States; 3 Rutgers University, Philadelphia, Pennsylvania, United States; 4 Drexel University, College of Nursing and Health Professions, Philadelphia, Pennsylvania, United States; 5 University of Pennsylvania, School of Nursing, philadelphia, Pennsylvania, United States

## Abstract

Recruitment of diverse community-dwelling persons living with dementia (PLWD) and their caregivers (dyads) into randomized controlled trials (RCT) is challenging, time consuming and expensive. This presentation will describe community outreach efforts used over a one-year period to recruit dyads of PLWD and their caregivers in Healthy Patterns RCT. Community outreach yielded 296 inquiries, such that people expressed interest in joining the study. Of the 296 inquiries, almost all (95.6%) identified as African American, and 91(30.7%) consented to join the study. Presentations at senior centers yielded the highest number of inquiries (n=148), followed by staff presence at various community events such as health fairs and senior galas (n=145) and referrals (n=3). We found that community outreach was an effective recruitment strategy to generate inquiries among diverse PLWD and their caregivers to enroll in Healthy Patterns. We will discuss these strategies and provide suggestions for recruiting diverse dyads into clinical trials.

